# Biomarkers in Acute Mesenteric Ischemia: A Systematic Review of Diagnostic Performance and Injury-Related Signals

**DOI:** 10.3390/diagnostics16203360

**Published:** 2026-10-15

**Authors:** Zelimkhan Berikkhanov, Zakhar Akulov, Ekaterina Klimova, David Pogosyan, Evgeniy Tarabrin, Alexey Shestakov, Aleksey Kotelnikov, Andrey Nikolaev, Vadim Razumovsky, Milena Ivanova, Mehrshad Ebrahimnezhad, Sara Nourmahal, Sergey Muraviev

**Affiliations:** 1 Department of Hospital Surgery No. 2, Sechenov First Moscow State Medical University ( Sechenov University), 119048 Moscow, Russia; berikkhanov_z_g@staff.sechenov.ru (Z.B.); klimova_e_a1@student.sechenov.ru (E.K.); david.pogos.41@gmail.com (D.P.); tarabrin_e_a@staff.sechenov.ru (E.T.); shestakov_a_l@staff.sechenov.ru (A.S.); kotelnikov_a_g@staff.sechenov.ru (A.K.); nikolaev_a_m@staff.sechenov.ru (A.N.); ivanova_m_yu_1@staff.sechenov.ru (M.I.); nurmakhal_s@student.sechenov.ru (S.N.); muravev_s_yu@staff.sechenov.ru (S.M.); 2 Cardiology Department No. 2, Yudin City Hospital of Moscow, The Patrice Lumumba Peoples’ Friendship University of Russia (RUDN), 117198 Moscow, Russia; mehrshad.ebrahimnejad@gmail.com

**Keywords:** acute mesenteric ischemia, biomarkers, diagnostic performance, I-FABP, citrulline, α-GST, SM22, urinary biomarkers, CT angiography

## Abstract

**Background/Objectives**: We examined nine biomarkers for acute mesenteric ischemia (AMI) detection, necrosis and postoperative prediction. **Methods**: PubMed/MEDLINE, the Cochrane Library and indexed citation links were searched on 10 October 2026 without a lower publication-year limit. PubMed and citation searches used an October 2026 publication cutoff. English- and Russian-language clinical reports with at least 10 participants were eligible. Narrative synthesis used QUADAS-2 and diagnostic GRADE. **Results**: Twenty-nine primary reports were included, with reported cohorts of 18–1479 participants. Cohort overlap and varying tested denominators precluded an unduplicated participant total. Among 28 diagnostic reports, 22 had high index-test risk and 25 had high flow/timing risk. Certainty was very low for eight markers. I-FABP, α-GST and citrulline findings depended on population, assay and timing. D-dimer, lactate, procalcitonin and ischemia-modified albumin lacked intestinal specificity. Urinary I-FABP estimates were imprecise. SM22 supplied association data without diagnostic-performance estimates. **Conclusions**: No evaluated marker supports reliable stand-alone exclusion of AMI or determination of bowel viability. Complementary panels require prospective validation. The injury diagrams are unvalidated research models.

## 1. Introduction

The diagnostic problem in acute mesenteric ischemia (AMI) is to recognize the threatened bowel while it remains viable. Early symptoms may be nonspecific, examination findings can underestimate injury, and advanced disease still carries substantial mortality despite access to revascularization and intensive care [1,2,3]. A useful biomarker would need to resolve uncertainty during this interval, when treatment can still preserve the bowel.

Computed tomography angiography (CTA) is the principal imaging test when AMI is suspected [4,5,6]. The difficulty often begins before imaging is requested. Abdominal pain, ileus, infection, and circulatory failure can lead to different initial diagnoses, particularly in patients with non-occlusive mesenteric ischemia. Biomarker research is therefore most clinically relevant when it addresses recognition in an undifferentiated patient, rather than merely separating established infarction from health [7,8].

Etiology, tissue viability, and systemic consequences describe different aspects of AMI. They should be recorded separately because neither symptom duration nor a biochemical concentration fixes the depth of injury. Table 1 summarizes these clinical dimensions. Chronic vascular symptoms require a separate diagnostic assessment [1,2,7,8,9].

Enterocyte injury releases intestinal fatty acid-binding protein (I-FABP), while α-glutathione S-transferase (α-GST) can reflect cellular damage in both intestinal and extraintestinal tissues. Citrulline relates to functional enterocyte mass. Human ischemia–reperfusion observations support the I-FABP injury signal [10]. Published syntheses show that apparent diagnostic performance changes with the assay and the definition of intestinal ischemia [11,12].

Smooth muscle protein 22 (SM22) addresses deeper mural injury, whereas ischemia-modified albumin (IMA) reflects a more general ischemic stress response. Citrulline and procalcitonin add functional and inflammatory information [13,14,15,16]. The clinical challenge is to establish what these signals add in a symptomatic patient, before a vascular cause or irreversible injury has been confirmed [17].

This review examines nine biomarkers against three clinical targets, namely AMI detection, necrosis and subsequent postoperative deterioration. Study-level findings are interpreted alongside a biological model of local injury, barrier disturbance and systemic response. The model defines questions for prospective testing and has no validated clinical thresholds.

## 2. Materials and Methods

### 2.1. Review Design and Reporting

This systematic review uses narrative synthesis. Reporting uses the Preferred Reporting Items for Systematic Reviews and Meta-Analyses (PRISMA) 2020 checklist, the PRISMA statement and the PRISMA-DTA extension for diagnostic test accuracy reviews [18,19,20]. A completed PRISMA 2020 checklist accompanies the manuscript (see Supplementary Materials), and the selection flow is presented in the main text. No review protocol was prepared, and the review was not registered in PROSPERO.

### 2.2. Information Sources and Search Strategy

PubMed/MEDLINE through NCBI E-utilities and the Cochrane Library through Search Manager were searched on 10 October 2026. Indexed backward and forward citation searches through PubMed ELink were conducted on the same date. PubMed and citation searches used a publication-date limit of October 2026. Cochrane records were restricted to publication years through 2025. No lower publication-year limit was imposed. The search combined controlled vocabulary and free-text terms for intestinal ischemia and the nine biomarkers. Full search expressions are given below.

The disease block covered mesenteric, intestinal and bowel ischemia, both ischemia and ischaemia spellings, infarction, necrosis, strangulation, non-occlusive disease and mesenteric venous thrombosis. The marker block included the principal names and aliases of all nine index tests. No diagnostic-performance, study-design, age or language filter was applied at retrieval. Indexed reference and citing links were retrieved through PubMed ELink for the 29 included primary reports and the systematic reviews by Treskes et al. and Reintam Blaser et al. [11,12]. ELink has incomplete citation coverage.

The PubMed/MEDLINE query was (“Mesenteric Ischemia”[Mesh] OR ((mesenteric[tiab] OR intestinal[tiab] OR bowel[tiab] OR splanchnic[tiab] OR gut[tiab]) AND (ischemi*[tiab] OR ischaemi*[tiab] OR infarct*[tiab] OR necros*[tiab] OR strangulat*[tiab])) OR NOMI[tiab] OR “mesenteric venous thrombosis”[tiab] OR “mesenteric vein thrombosis”[tiab]) AND (“Fatty Acid-Binding Proteins”[Mesh] OR “I-FABP”[tiab] OR IFABP[tiab] OR FABP2[tiab] OR “intestinal fatty acid binding protein”[tiab] OR “intestinal fatty acid-binding protein”[tiab] OR “Glutathione Transferase”[Mesh] OR “alpha glutathione S transferase”[tiab] OR “alpha glutathione S-transferase”[tiab] OR “alpha-GST”[tiab] OR “alpha GST”[tiab] OR “α-GST”[tiab] OR “Fibrin Fibrinogen Degradation Products”[Mesh] OR “D-dimer”[tiab] OR “D dimer”[tiab] OR “Lactic Acid”[Mesh] OR lactat*[tiab] OR “Citrulline”[Mesh] OR citrullin*[tiab] OR “Procalcitonin”[Mesh] OR procalcitonin[tiab] OR SM22[tiab] OR “SM22-alpha”[tiab] OR “SM22α”[tiab] OR transgelin[tiab] OR TAGLN[tiab] OR “ischemia modified albumin”[tiab] OR “ischemia-modified albumin”[tiab] OR “ischaemia modified albumin”[tiab] OR “ischaemia-modified albumin”[tiab] OR “albumin cobalt binding”[tiab] OR “cobalt albumin binding”[tiab] OR IMA[tiab]) AND (“1800/01/01”[Date—Publication]:“2026/10/10”[Date—Publication]).

The Cochrane Search Manager strategy used the following lines.

#1 MeSH descriptor: [Mesenteric Ischemia] explode all trees.

#2 (((mesenteric OR intestinal OR bowel OR splanchnic OR gut) AND (ischem* OR ischaem* OR infarct* OR necros* OR strangulat*)) OR NOMI OR “mesenteric venous thrombosis” OR “mesenteric vein thrombosis”):ti,ab,kw.

#3 (“fatty acid binding protein” OR “fatty acid-binding protein” OR “I-FABP” OR IFABP OR FABP2 OR “glutathione transferase” OR “glutathione S-transferase” OR “alpha GST” OR “alpha-GST” OR “D-dimer” OR “D dimer” OR lactate OR “lactic acid” OR citrulline OR procalcitonin OR SM22 OR transgelin OR TAGLN OR “ischemia modified albumin” OR “ischemia-modified albumin” OR “ischaemia modified albumin” OR “ischaemia-modified albumin” OR “albumin cobalt binding” OR “cobalt albumin binding” OR IMA):ti,ab,kw.

#4 (#1 OR #2) AND #3.

### 2.3. Eligibility Criteria

The clinical question concerns biomarkers in adults with suspected or confirmed vascular AMI. The nine index tests are I-FABP, α-GST, D-dimer, L-lactate, D-lactate, citrulline, IMA, procalcitonin and SM22 in blood or urine. Clinical cohort, cross-sectional and case–control reports are eligible when they provide biomarker data relevant to AMI or intestinal injury. AMI detection, discrimination of necrosis and prediction of later events are separate targets. Confirmation may use CTA, catheter angiography, endoscopy, operation, histology, autopsy or a defined clinical reference. These reference standards are assessed individually rather than treated as interchangeable.

The eligibility scope includes English- and Russian-language clinical reports with at least 10 participants and relevant biomarker data. Mixed-age cohorts and reports dominated by obstruction or colitis provide indirect evidence for the adult vascular-AMI question. The SM22 association report is considered separately from diagnostic-performance studies. Reviews, guidelines, protocols and experimental models provide context and are excluded from the primary diagnostic evidence. Reports without an eligible biomarker or clinical outcome, nonclinical studies and reports failing the language or sample-size criteria were excluded from the primary evidence. Published articles were the report type used, with indexed primary data retained when full-text access was incomplete.

### 2.4. Study Selection

Two researchers independently screened the titles and abstracts of deduplicated records and assessed the retrieved reports against the eligibility criteria. Duplicate records were identified by PMID and study metadata. Final eligibility decisions were agreed between the two researchers. Records are bibliographic search entries, whereas reports are the publications assessed for eligibility. Multiple reports from one cohort were linked during extraction.

**Figure 1:**
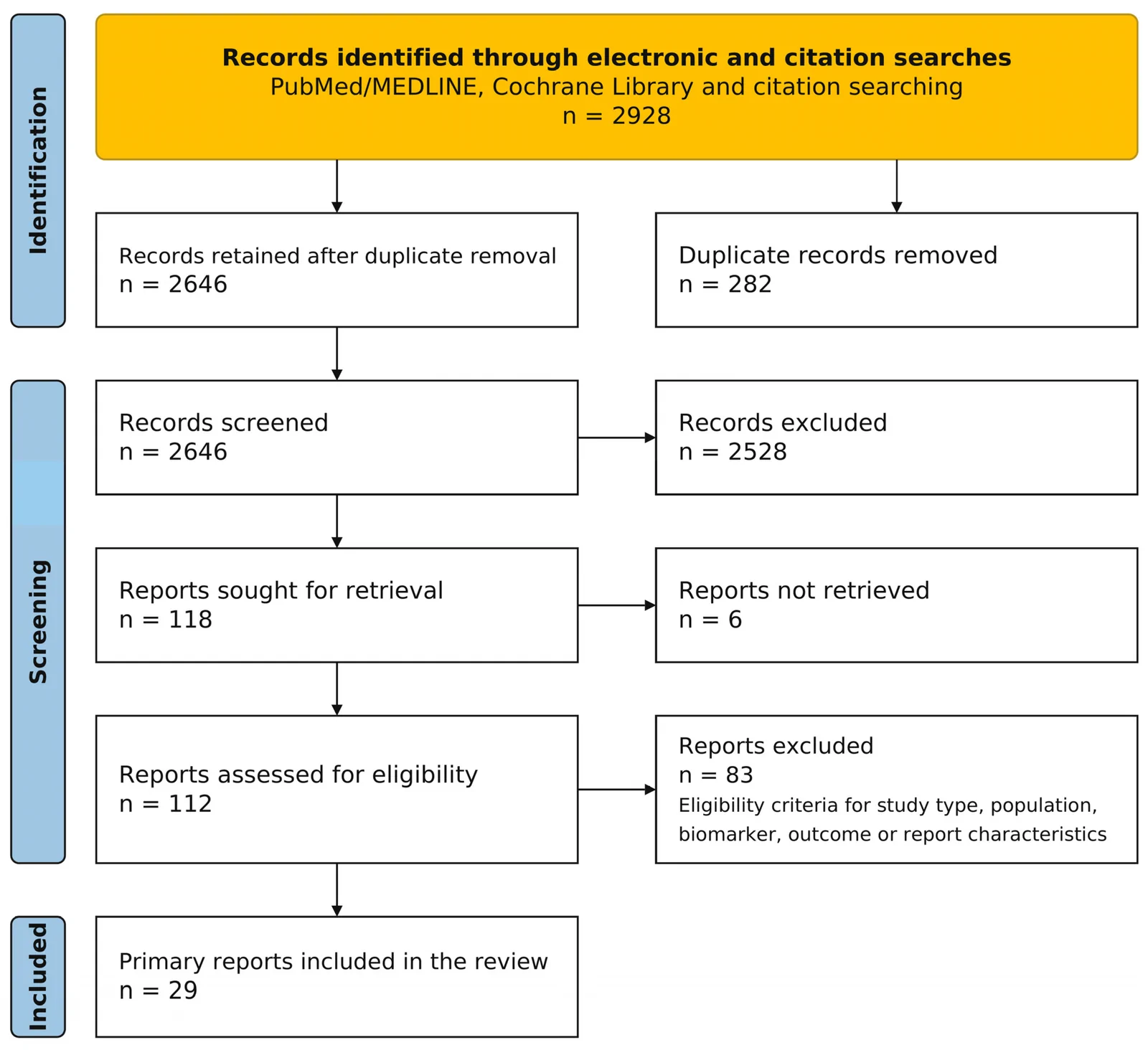
PRISMA 2020 flow diagram of study identification and selection. Identification totals combine electronic and indexed citation searches. Counts distinguish records from reports. Included reports are not necessarily independent cohorts.

Figure 1 shows the search and selection sequence.

### 2.5. Data Extraction and Outcomes

Two researchers (S.N. and M.I.) independently extracted data from each included report. Data extraction recorded author, year, country, design, tested sample size, setting, etiology, severity, specimen, assay, threshold, sampling time and reference standard. Sensitivity, specificity, area under the receiver operating characteristic curve (AUC), confidence intervals and predictive values were recorded when available. The accessible source set comprised 28 full reports and one indexed primary abstract. Unreported items were recorded as not reported or unclear. Detection refers to AMI or intestinal ischemia at the assessment time, necrosis refers to independently established irreversible or transmural injury, and postoperative prediction refers to a later ischemic event.

Results from different thresholds, specimens and time points were not combined. The study-level table presents the principal reported comparison for the clinical target, with alternative results discussed when they affect interpretation. Missing values were not imputed. A 2 × 2 table was retained only when explicitly published or uniquely reconstructable from stated denominators and results, with reconstruction labeled. Conflicting source values were annotated without selecting a more favorable estimate. Confirmed cohort overlap was recorded and overlapping reports were not treated as independent. No primary-study authors were contacted. Sensitivity and specificity are expressed as percentages, AUC is dimensionless, and reported association estimates are distinguished from diagnostic classification. Published confidence intervals are reproduced without recalculation.

### 2.6. Risk of Bias and Applicability

Two researchers independently assessed 28 diagnostic reports with QUADAS-2 using four risk-of-bias and three applicability domains [21]. The 11 signaling questions addressed patient selection, index-test conduct, the reference standard and flow/timing. Judgments used the available source information, with unclear ratings where reporting was insufficient. The target question was vascular AMI in adults at clinical suspicion. Studies of necrosis and postoperative prediction were interpreted against their own targets. In multi-test reports, prespecified and exploratory thresholds were distinguished. SM22 was outside diagnostic-performance assessment because no classification estimate was reported. Disagreements were resolved by consensus. No numerical quality score was calculated.

### 2.7. Synthesis and Certainty of Evidence

Studies were synthesized by marker, specimen and target condition. No new meta-analysis or quantitative subgroup analysis was performed. The synthesis separates initial detection, necrosis and subsequent postoperative events because these estimates concern different clinical questions. Assays, thresholds, sampling times, reference standards and cohort overlap also differ. Heterogeneity alone does not rule out meta-analysis of a sufficiently defined, compatible subset. Published pooled estimates are presented as secondary evidence and may include additional reports or overlapping cohorts. Study characteristics and diagnostic results were tabulated in publication-year order. Risk-of-bias judgments were displayed for each report and as domain counts, and certainty was summarized in a GRADE table. Heterogeneity was examined descriptively across patient selection, AMI subtype and severity, reference standard, assay and specimen, sampling time and diagnostic threshold. No meta-regression or formal sensitivity analysis was conducted.

Diagnostic GRADE was applied to defined clinical questions [22]. A potentially high-certainty diagnostic evidence body was assessed for risk of bias, inconsistency, indirectness, imprecision and publication bias. Downgrades of one or two levels were documented, with certainty bounded at very low. Observational design alone did not determine the starting certainty. Report counts were retained without summing participants across overlapping cohorts or repeated measurements. SM22 received no diagnostic rating because it lacked a diagnostic-performance estimate. Publication bias was undetermined and incurred no automatic downgrade. Diagnostic discrimination was assessed separately from clinical benefit. No formal assessment of bias due to missing results, funnel-plot analysis or test for small-study effects was performed. Target and threshold incompatibility, small evidence groups and cohort overlap limited the interpretation of such tests.

### 2.8. Ethics

The review analyzed published literature and did not collect new patient data or conduct animal experiments. Institutional review board approval and informed consent were therefore not applicable.

## 3. Results

### 3.1. Study Selection and Clinical Context

The searches yielded 2928 records. After removal of 282 duplicates, 2646 records underwent title and abstract screening, and 2528 were excluded. Of 118 reports sought for retrieval, six could not be retrieved. The remaining 112 reports were assessed for eligibility, and 83 were excluded. Twenty-nine primary publications were included, comprising 17 published from 2015 onwards and 12 before 2015. The search had no lower publication-year limit. All 29 included reports were identified by the documented PubMed query. Reports were excluded for ineligible study type or population, absence of relevant biomarker or outcome data, or failure to meet the language or minimum sample-size criteria described in Section 2.3.

Table 2 identifies every primary report, its population, test, clinical target and principal result. These are 29 publications rather than 29 independent cohorts. Matsumoto 2019 explicitly extends the 2014 cohort, while overlap between some postoperative reports cannot be excluded [23]. Patient selection, target definitions and reporting limitations are retained for each study.

The human ischemia–reperfusion report [10] was excluded from the primary diagnostic synthesis because it studied experimentally induced injury rather than suspected clinical AMI. Reviews and meta-analyses [11,12,15,16,17] were excluded from primary evidence because they did not provide independent primary observations. They were retained for background and secondary interpretation.

### 3.2. Risk of Bias and Applicability

Among 28 diagnostic reports, patient-selection risk was high in 15, low in six and unclear in seven. Index-test risk was high in 22, low in one and unclear in five. Reference-standard risk was high in four, low in three and unclear in 21. Flow/timing risk was high in 25 and low in three. No report was low risk in all four domains. High applicability concern affected 25 reports for the population, five for index-test conduct and 18 for the target/reference definition. Table 3 gives the complete report-level results, including the separately identified SM22 association report.

**Figure 2:**
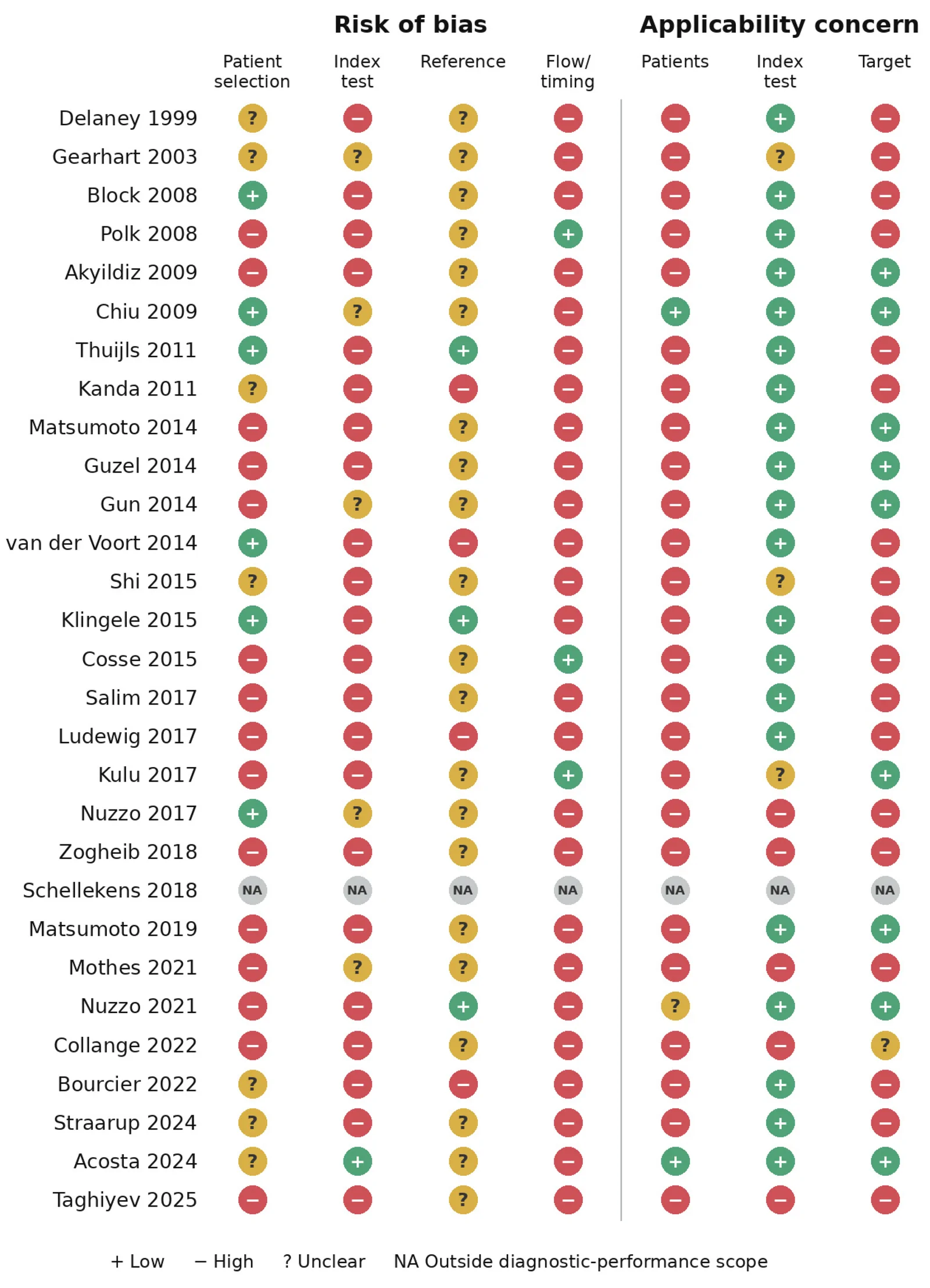
Report-level QUADAS-2 risk of bias and applicability. Judgments reflect source reporting and relevance to suspected vascular AMI in adults. S21 is outside the diagnostic-performance assessment [23,24,25,26,27,28,29,30,31,32,33,34,35,36,37,38,39,40,41,42,43,44,45,46,47,48,49,50,51].

Figure 2 displays the individual domain judgments, with the non-diagnostic SM22 report identified separately.

**Figure 3:**
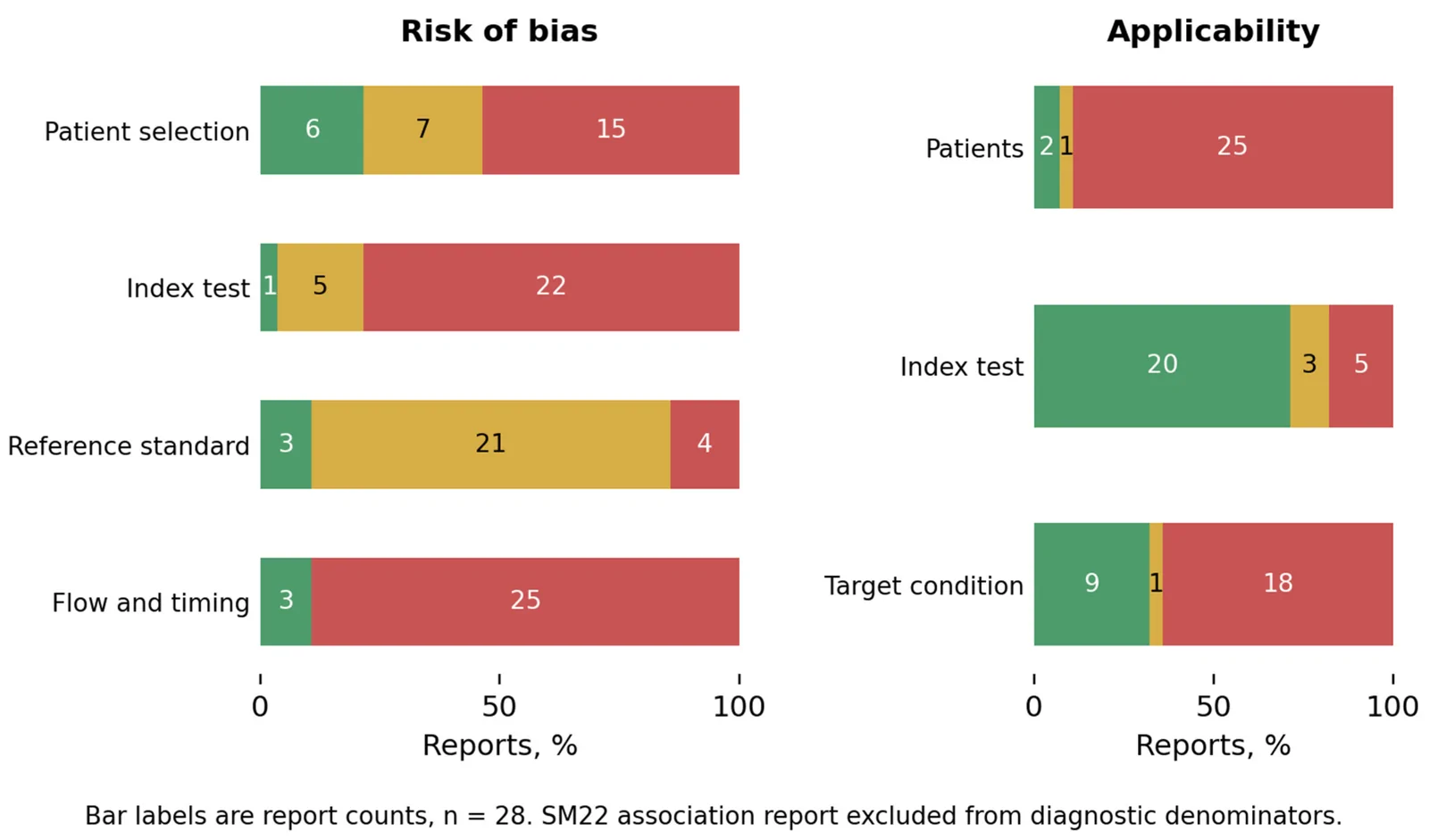
QUADAS-2 domain distributions. Green, low. Yellow, unclear. Red, high. Numbers within bars are counts, with *n* = 28 in each diagnostic domain. The SM22 association report is omitted from these denominators.

Figure 3 summarizes risk and applicability across the 28 diagnostic reports.

### 3.3. Enterocyte Injury and Functional Mass

I-FABP findings depend on which injury is being detected. Kanda analyzed 361 acute-abdomen patients, including children and 52 small-bowel ischemia cases dominated by strangulation. At 3.1 ng/mL, sensitivity was 78.8% and specificity 73.8%. The published AUC of 0.792 came from the surgical subset in which the threshold was developed, not from an independent validation cohort [31]. Matsumoto reported more favorable discrimination for vascular disease and NOMI, but both reports selected patients after clinical/CT assessment and partly reused one cohort [23,32].

More direct vascular evidence was less favorable. In Nuzzo 2021, with 50 arterial/venous AMI cases and 79 symptomatic controls, the source performance table reported I-FABP AUC 0.401 [46]. Bourcier studied necrosis in severe ICU NOMI rather than initial detection, with AUC 0.83 in 40 classifiable patients [48]. Taghiyev reported a high 36 h postoperative estimate based on six confirmed events, but its two ROC tables give discordant AUCs for the same named time points [51]. These results cannot be reconciled by treating I-FABP as a uniform test with one threshold.

α-GST illustrates the same problem. Delaney reported sensitivity of 100% at 4 ng/mL in 26 patients, whereas Block reported 20% at that cutoff in a broader cohort [24,26]. Gearhart reported 74% overall correct classification and improved performance when α-GST was combined with other tests [25]. Citrulline was insensitive in the operative Kulu comparison, modestly discriminative in Nuzzo 2021 and nondiscriminative in severe ICU NOMI [41,46,48]. Renal metabolism and the distinction between enterocyte function and an acute vascular event remain central to interpretation [14,15].

### 3.4. Coagulation, Metabolic, and Inflammatory Signals

D-dimer is particularly dependent on the clinical spectrum and threshold. Chiu reported sensitivity of 96% and specificity of 18% at an existing hospital cutoff of 1.0 μg FEU/mL, with one missed AMI case [29]. In Acosta, all 23 tested MVT cases exceeded their local normal range, but specificity was 20.6% among 34 tested controls. D-dimer was available in only 57 of 360 patients, and the separate ROC-optimal 5 mg/L cutoff did not produce that sensitivity/specificity pair [50]. Published syntheses likewise give different pooled estimates [52,53]. None validates a universal negative-test pathway for early AMI.

L-lactate describes metabolic disturbance but did not discriminate AMI well in matched ICU patients. Collange reported AUC 0.57 during the first 24 h and 0.60 during the next 24 h [47]. Mothes evaluated later postoperative ischemia, while Nuzzo 2017 evaluated irreversible necrosis among patients already known to have AMI [42,45]. Their endpoints differ from initial diagnosis. Experimental lactate findings are mechanistic context [54]. A normal lactate result must not defer indicated CTA [2].

D-lactate likewise performed poorly in more recent clinical comparisons. Straarup reported sensitivity of 26.1% after excluding half the enrolled samples for lipemia. Only six target-positive patients had vascular necrosis, and four other positives had reversible nonvascular ischemia [49]. Nuzzo reported a D-lactate AUC of 0.438 [46]. Earlier syntheses combined broader intestinal-ischemia populations [11,55]. Their more favorable estimates do not establish an emergency rule-out test.

Procalcitonin results also require an outcome-specific reading. Klingele evaluated subsequent angiographic NOMI after cardiac surgery, with selective angiography [37]. Cossé compared necrosis with nonnecrotic injury among 128 operated ischemic patients, not AMI with symptomatic non-AMI [38]. Zogheib developed the postoperative PALM score in 144 patients. Bootstrap resamples described as a 1000-patient population were internal resampling, not 1000 additional patients [43]. The broader PCT literature includes ischemic and obstructive conditions with substantial inflammatory confounding [16].

### 3.5. IMA and SM22

Polk reported favorable IMA discrimination in 26 operative patients at a threshold derived in that same sample [27]. Small earlier clinical series and their pooled estimates provide additional context but not independent validation of this threshold [11,13]. The time-course paper by Gündüz used rats and cannot establish human kinetics [56]. Extraintestinal ischemic stress and assay dependence prevent organ-specific interpretation.

The SM22 report compared 13 transmural and nine mucosal ischemia cases with separate symptomatic and healthy groups. Plasma SM22 was higher in transmural injury, but no clinical cutoff, sensitivity, specificity or ROC estimate was reported [44]. This is evidence of an association to test, not a validated depth classifier or resection criterion.

### 3.6. Performance Estimates and Biological Characteristics

Table 4 places the primary findings alongside published diagnostic syntheses. These pooled values come from separate evidence sets. Treskes reported assay-specific estimates [11]. Reintam Blaser reported serum I-FABP sensitivity of 73.9% and specificity of 90.5% across 16 studies [12]. The secondary estimates provide context for the study-level findings in Table 2.

Table 5 combines molecular properties, specimens and analytical methods with candidate signals of mucosal injury, enterocyte function, inflammation and deeper mural injury. These processes overlap and do not define validated stages or diagnostic thresholds.

**Figure 4:**
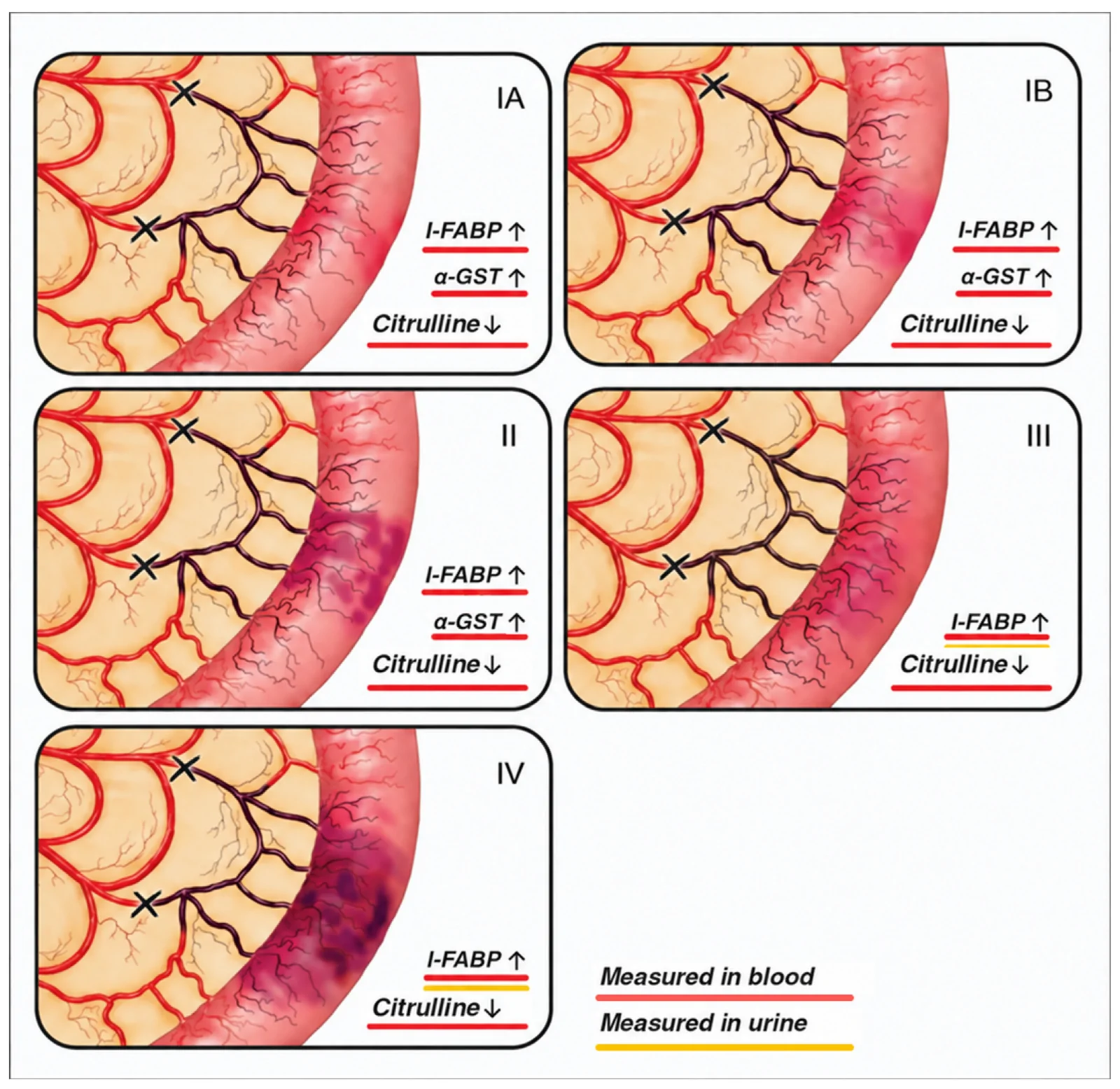
Conceptual intestinal-wall injury model. Red underlining denotes blood measurements and yellow underlining denotes urine measurements. Crosses indicate interrupted vascular flow. Upward and downward arrows indicate hypothesized increases and decreases in biomarker concentrations. Serial I-FABP and α-GST measurements could test mucosal injury, while citrulline trajectories could test functional loss or recovery. Interpretation requires defined sampling times and assessment of clearance and extraintestinal sources. Panels (**IA**–**IV**) and the depicted trajectories are hypothesis-generating and unvalidated. The model has no established role in clinical staging or treatment.

Figure 4 links the intestinal-wall injury model to prospective questions about mucosal damage and recovery of enterocyte function. I-FABP is shown in panels IA and IB to reflect its possible release during mucosal injury [10]. IA–IV are descriptive labels for the model and do not define clinical stages.

**Figure 5:**
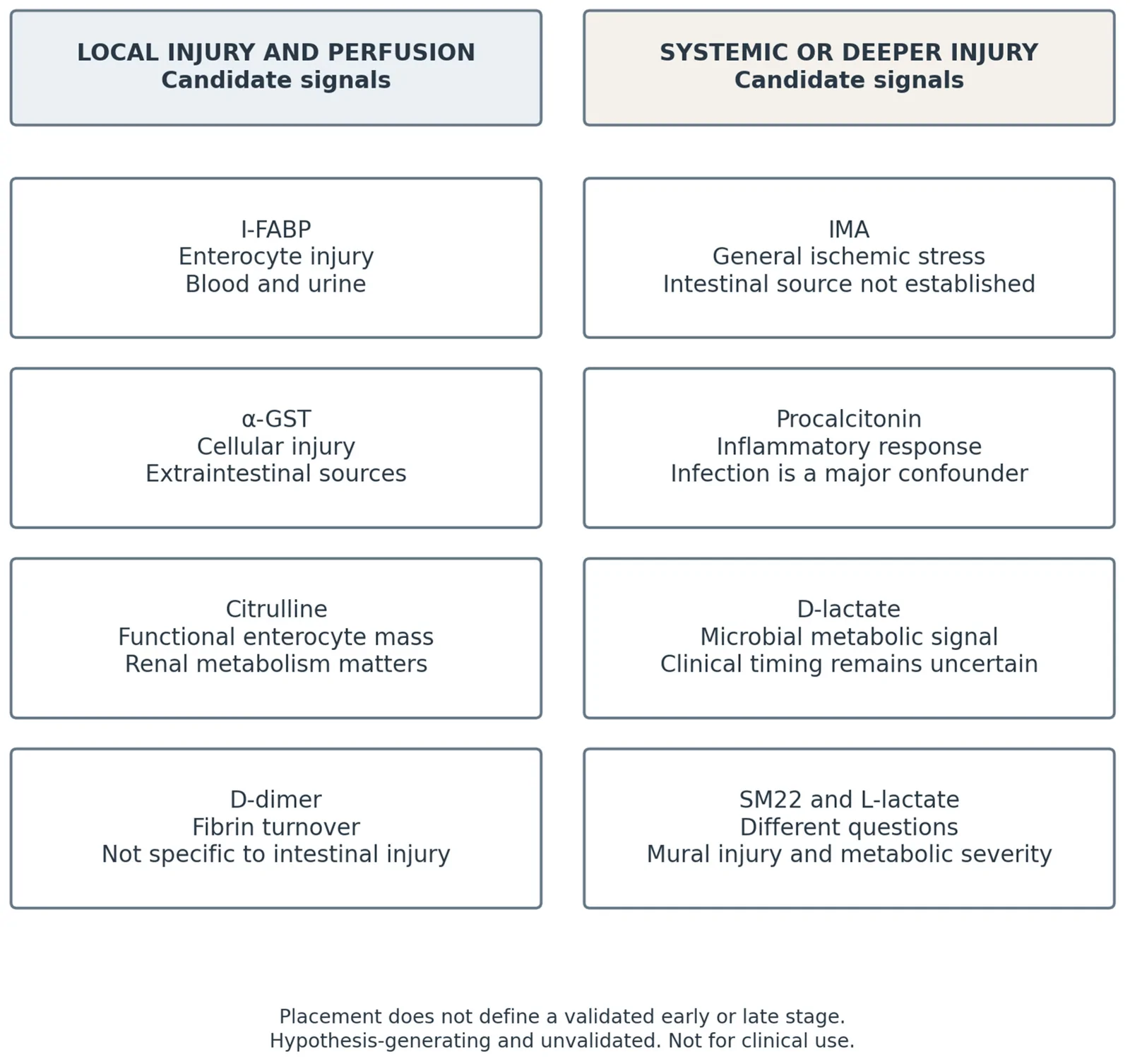
Candidate signals during intestinal injury. The arrangement is hypothesis-generating and unvalidated. Study-specific diagnostic estimates appear in Table 2 and cannot be assigned to the illustrated injury phases.

Figure 5 distinguishes candidate signals of local injury from systemic or deeper injury. Their placement indicates hypotheses to test, without stage-specific sensitivity or specificity.

### 3.7. Certainty and Clinical Applicability

Diagnostic certainty was very low for blood and urinary I-FABP, α-GST, citrulline, D-dimer, L-lactate, D-lactate, procalcitonin and IMA. Selection and verification problems, optimized thresholds, target mismatch and imprecision drove the downgrades. SM22 had no diagnostic estimate to grade. Table 6 presents the domain judgments. Bias due to missing results remained undetermined for the diagnostic evidence groups because no formal reporting-bias assessment was undertaken.

The blood I-FABP evidence combines mixed etiologies, CT-selected cohorts and postoperative sampling. Some estimates describe necrosis rather than early vascular AMI. Assay differences and thresholds derived in the tested cohort limit transferability [23,30,31,32,46,48,51]. Urinary I-FABP adds substantial imprecision, with only 19, 18 and 36 tested participants in the three cohorts and missing specimens in critically ill patients [30,39,40].

For α-GST, sensitivity was 100% in Delaney and 20% in Block at 4 ng/mL [24,26]. Citrulline estimates depend on population, renal metabolism and analytical method [41,46,48]. Selected patients, optimized cutoffs and unequal verification support downgrades for bias, inconsistency and indirectness.

D-dimer studies combine different etiologies and assay limits, with selective testing in some larger cohorts [34,50]. L-lactate and procalcitonin often address necrosis or postoperative ischemia rather than early AMI detection [37,38,42,43,45,47]. D-lactate is affected by reporting discrepancies and large exclusions for assay interference [36,46,49]. These sources of bias and target indirectness underlie the certainty judgments.

IMA rests on 26 operative patients and a derived threshold, which caused a very serious downgrade for imprecision [27]. Inconsistency cannot be graded from one primary report. No diagnostic-classification body exists for SM22 [44].

Very low certainty denotes substantial uncertainty in a transferable diagnostic estimate. It is not evidence that a marker has no biological association. Clinical utility requires additional evidence that a testing strategy improves decisions or outcomes. Such benefit was not demonstrated by the reviewed reports.

## 4. Discussion

### 4.1. Clinical Question and Contribution

A biomarker can track intestinal injury yet fail at the decision point. An early-AMI test must discriminate among plausible causes of abdominal pain, retain sensitivity before extensive necrosis and return soon enough to influence care. Healthy-control comparisons, histological necrosis and future postoperative events address different questions [17,52,57]. Reintam Blaser synthesized a broad range of biomarkers and AMI types, whereas Blauw focused on arterial occlusive disease [12,17]. Here, nine specified markers are examined at the study level, with detection, necrosis and postoperative prediction kept separate. Recent venous and postoperative reports add clinical settings in which transferability of earlier estimates can be examined.

Three questions guide interpretation. Is intestinal injury present? How deep or extensive is it? Does serial change inform progression or recovery? Each requires a defined population, reference standard, sampling schedule and endpoint. Better discrimination alone does not demonstrate fewer missed diagnoses, shorter treatment delays or less bowel loss.

### 4.2. Relating a Laboratory Signal to Tissue Injury

The proposed sequence begins with epithelial injury, extends to barrier disruption and inflammatory signaling, and may culminate in deeper mural injury and organ dysfunction. These processes can overlap. Reperfusion, disease mechanism, collateral flow, renal function, and treatment may alter both their order and the measured concentrations. The scheme therefore offers a biological rationale for serial measurement without defining a fixed clinical clock.

Serial I-FABP and α-GST measurements could test whether an injury signal precedes systemic deterioration, while citrulline trajectories could describe loss or recovery of enterocyte function. Renal metabolism, extraintestinal sources and sampling time require explicit assessment. None identifies a vascular cause by concentration alone, and favorable findings in strangulation or healthy-control comparisons should not be recast as validated AMI detection [26,31,33,41]. SM22 should be tested against independently determined injury depth to establish whether a smooth-muscle signal distinguishes transmural from non-transmural injury [44].

Serial D-lactate and procalcitonin measurements could test whether barrier-associated and inflammatory signals accompany progression or recovery. Infection, microbial production and clearance may confound these trajectories. D-dimer and L-lactate should be tested for added severity information beyond clinical and imaging assessment, with shock and coagulation recorded as covariates. IMA requires comparison with extraintestinal ischemia rather than a healthy reference alone [27,47].

### 4.3. Recognizing an Acute Presentation

Acute and chronic mesenteric ischemia require separate interpretation. Postprandial symptoms and weight loss warrant vascular assessment in their clinical context [4,9]. Sudden or worsening pain, circulatory deterioration, or other findings that raise suspicion of AMI require an urgent pathway [1,2]. Routine biomarkers cannot safely arbitrate between these pathways.

### 4.4. Clinical Interpretation and Practical Availability

A result is useful only if its meaning and limitations are understood. Table 7 distinguishes the information a marker might provide from decisions it cannot currently support.

#### 4.4.1. Routine Tests and Research Assays

D-dimer, L-lactate, and procalcitonin can be measured by routine clinical platforms, although local access and turnaround differ. Their availability does not create an AMI-specific indication. Conversely, measuring citrulline is technically established in specialist amino-acid testing, including a clinical reference-laboratory panel [58], but that does not make it an emergency test for AMI. Table 8 separates analytical access from clinical validation.

#### 4.4.2. Urinary Sampling

Urinary I-FABP has been assessed in three small clinical cohorts. Thuijls reported sensitivity/specificity of 90/89% in only 19 paired urine participants. Salim reported 92/80% in 18 patients, including five nonischemic controls. Ludewig reported 81.3/70% in 36 ICU patients, with missing samples in renal/anuric patients [30,39,40]. These exploratory cutoffs differ, and the strongest estimate cannot be assumed to transfer across populations or assays.

Urine is useful for repeated sampling only when collection is prompt and renal handling is documented. Anuria may remove the sickest patients from the urine analysis, while dilution, acute kidney injury and renal replacement can alter concentrations. Studies should pair blood and spot urine, record actual collection times, urine output and creatinine, and prespecify how dilution is handled. Creatinine normalization needs validation during acute kidney injury. Waiting for a 24 h collection is incompatible with urgent AMI assessment.

Prostaglandin E-major urinary metabolites (PGE-MUM) are a separate preclinical candidate. Suzuki examined them in a rat occlusion/reperfusion model [61]. That result does not establish human diagnostic performance. Proteinuria, pH and sediment abnormalities have no demonstrated intestinal specificity in the reviewed evidence. They should not be added to an AMI algorithm on the strength of plausible biology alone.

#### 4.4.3. Barriers to Implementation

A marker measured in stored samples may show a reproducible association yet remain impractical for emergency testing. Table 9 links the main implementation barriers to the evidence needed to address them.

### 4.5. Research Algorithms and Clinical Decisions

The proposed algorithms place biomarker sampling alongside standard assessment. Their value would depend on whether serial results improve a defined decision without increasing time to diagnosis or treatment. The imaging pathway is determined by clinical suspicion, not by a biomarker result [2].

**Figure 6:**
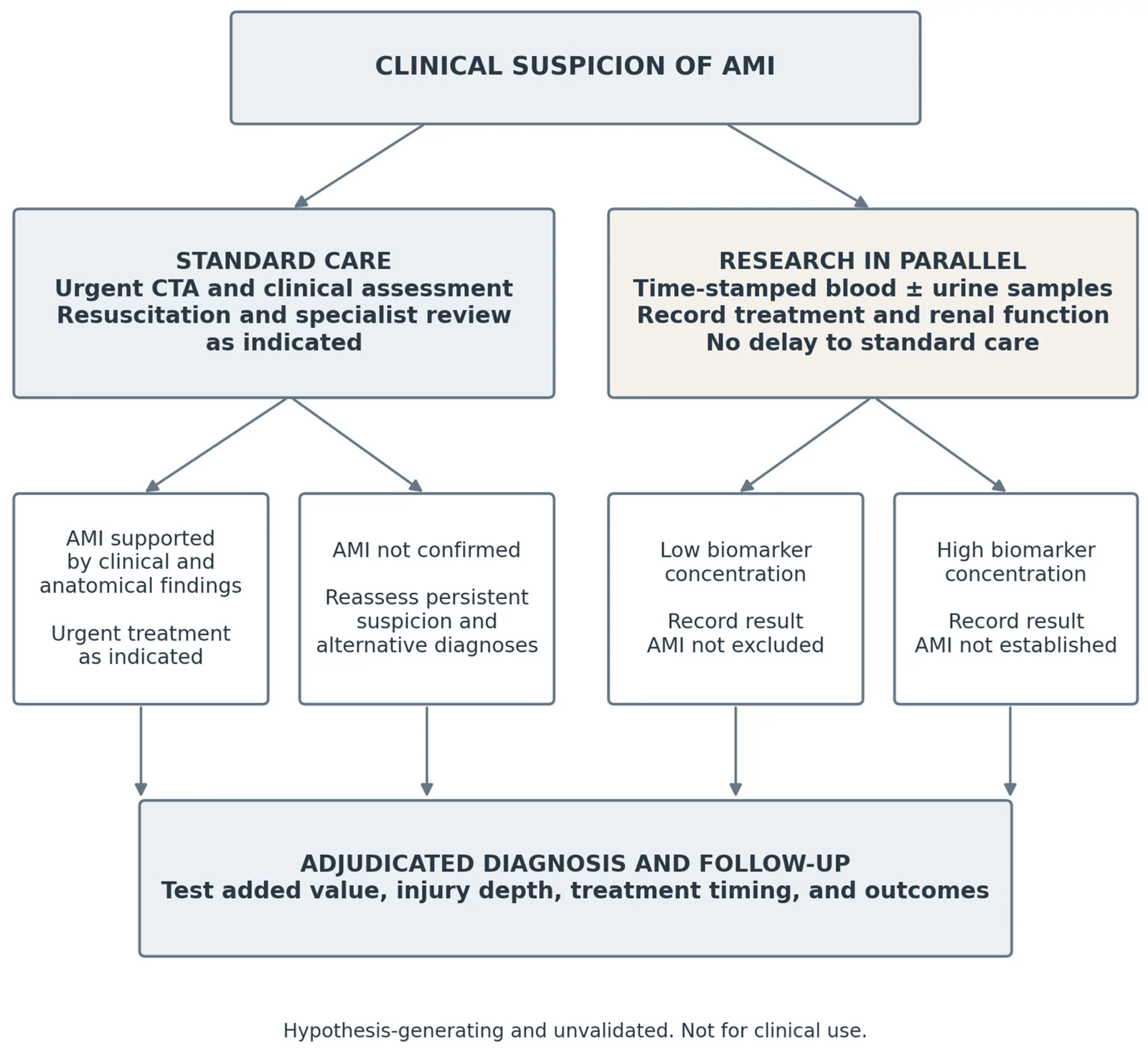
Research sampling alongside assessment of suspected AMI. Biomarker results do not determine access to CTA or intervention. This hypothesis-generating framework is unvalidated and is not a clinical algorithm.

Figure 6 places research sampling alongside the urgent AMI pathway. Positive and negative results are recorded for analysis while diagnosis and treatment proceed according to clinical and imaging findings.

### 4.6. Limitations and Sources of Heterogeneity

The most consequential heterogeneity concerns the question each study actually asks. Detecting any intestinal ischemia differs from identifying early vascular AMI, and both differ from detecting transmural necrosis. Table 10 sets out six dimensions that affect interpretation.

The absence of a prespecified protocol and PROSPERO registration limits assessment of selective eligibility, outcome definitions and analysis. The appraised set contains 12 publications before 2015, three mixed-age cohorts and one SM22 association report. Publication year does not limit eligibility, but the mixed populations and non-diagnostic target reduce directness for adult vascular AMI.

Search coverage was limited to two electronic sources and indexed citation links. Incomplete or conflicting source reports may affect individual QUADAS-2 judgments. Confirmed or possible cohort overlap prevents treating every report as an independent population. Language restrictions and undetermined publication bias further limit generalizability.

GRADE judgments concern specific diagnostic questions, not proof that testing improves outcomes. Published pools may include studies outside this report set and overlap each other. The tissue-stage diagrams derive from biological reasoning, without prospective patient-level validation in this review. Neither a diagram nor a marker combination establishes reversibility, bowel viability or clinical benefit.

### 4.7. Priorities for Prospective Research

#### 4.7.1. Why Evidence for New Markers Remains Limited

High-quality biomarker studies are difficult because the relevant sample must be obtained early, while urgent treatment takes priority. Disease mechanisms differ, advanced cases are easier to confirm, and uncomplicated noncases may never undergo surgery. The practical consequences for research design are summarized in Table 11.

#### 4.7.2. Rational Biomarker Panels

Panels should add information that individual markers and routine clinical assessment do not already provide. Kulu combined citrulline, D-dimer and lactate in 48 patients, while Zogheib developed a postoperative score in 144. Both models require independent validation [41,43]. Matsumoto found a modest increase in clinical-model AUC after adding I-FABP [23]. Thuijls found no improvement from adding correlated FABPs [30]. Panel size therefore needs a biological and statistical rationale.

One design would pair an enterocyte-injury marker with systemic context and, for a prespecified viability question, a candidate of deeper mural injury. Citrulline is more naturally tested against function or recovery. Barrier-associated or endothelial variables require a named analyte and their own validation, rather than inference from multiorgan failure. Renal function, shock and treatment should enter the analysis plan as confounders. A limited prespecified model should be compared with the same clinical model without the new markers.

#### 4.7.3. Design Principles

1. Register the prospective clinical study in an appropriate clinical-study registry and publish the protocol and analysis plan before enrollment or outcome analysis. Prespecify the clinical decision and primary endpoint.
2. Enroll consecutive patients at the point of suspected AMI and retain symptomatic non-AMI comparators. Document exclusions and missed eligible patients.
3. Collect serial samples at defined clinical time points, including presentation and before and after intervention when feasible. Record symptoms, resuscitation, renal function, and sample processing.
4. Separate arterial embolism, arterial thrombosis, venous thrombosis, and non-occlusive disease. Distinguish non-transmural injury from transmural necrosis using predefined adjudication.
5. Specify the assay and model in advance, control the number of predictors, and validate thresholds or model coefficients in an independent cohort.
6. Test incremental value beyond information available at the intended decision point. A model intended before CTA should be compared with a clinical model. A post-imaging model may also include CTA findings. Report discrimination, calibration, and net benefit.
7. Evaluate time to diagnosis and treatment, revascularization, resection extent, complications, and survival. Use these outcomes to determine whether the testing strategy merits a subsequent impact study.

#### 4.7.4. BIPAMI and TACTIC

The BIPAMI protocol specifies planned enrollment of 250 adults with suspected AMI, with centralized assays and separate comparisons of AMI detection and transmural injury. Sampling starts at admission or suspicion and follows the clinical scenario, including intervention and 4–6 h and 12 h afterwards, with sampling at reintervention when applicable [59]. This design can examine reperfusion separately from ongoing ischemia, although the published protocol does not establish performance.

The TACTIC protocol specifies planned enrollment of approximately 120 adults, with serial I-FABP, villin-1 and SM22 in blood and volatile organic compounds in exhaled breath. Clinical, imaging, operative and histological findings support diagnosis and injury characterization [60]. Both protocols address time and depth explicitly. Protocol publications describe the designs and intended sample sizes. They do not supply outcome estimates or validate the present diagrams.

#### 4.7.5. Integrating Clinical, Biological and Anatomical Evidence

A useful research model would connect the clinical decision point with enterocyte injury, systemic context and anatomical findings. Vascular risk and symptoms establish suspicion. Local markers provide a candidate tissue signal, while systemic measurements describe shock, coagulation and inflammation. Imaging and operative findings establish vascular anatomy and injury. Serial sampling can test whether the laboratory information improves this assessment. The model proposes no validated thresholds or numerical score.

## 5. Conclusions

The 29 appraised primary reports do not support a biomarker-only pathway for excluding AMI or determining bowel viability. Populations and outcomes differ, and some reports reuse cohorts. Diagnostic certainty is very low where performance evidence can be graded. SM22 remains an unvalidated injury-depth association. Blood and urine results require interpretation against the assay, sampling time, renal function and clinical target.

Prospective studies should test small complementary panels alongside standard care, with defined outcomes, serial sampling and external validation. Clinical benefit must be measured rather than inferred from an AUC. The proposed diagrams define research questions. Biomarker testing must not delay indicated CTA or urgent treatment.

## Figures and Tables

**Table 1:** Clinical dimensions of acute mesenteric ischemia.

Dimension	Categories or Features	Interpretation
Acute etiology	Arterial embolism, arterial thrombosis, mesenteric venous thrombosis, and non-occlusive mesenteric ischemia	Mechanisms require separate analysis in biomarker studies.
Tissue injury	Mucosal injury, deeper mural injury, and transmural necrosis	Biological distinctions. Biomarker thresholds for these categories are not validated.
Perfusion and clinical consequences	Compensated perfusion, persistent hypoperfusion, infarction, and peritonitis	Progression depends on collateral supply, mechanism, and treatment. Fixed hour-based stages are unreliable.

AMI, acute mesenteric ischemia. These dimensions do not constitute a biomarker-based staging scale. Sources are cited in the preceding paragraph.

**Table 2:** The 29 appraised primary reports and their diagnostic context.

Report/Country	Design, Analyzed Population	Test, Matrix and Assay	Sampling and Reference	Principal Diagnostic Result
S01. Delaney 1999 [24], Ireland	Prospective, *n* = 26. 12 intestinal ischemia, six vascular and six global.	Plasma α-GST, Biotrin ELISA.	Initial assessment. Operation, imaging, autopsy or recovery.	>4 ng/mL. Se/Sp 100/86%. Derived cutoff.
S02. Gearhart 2003 [25], USA	Prospective, *n* = 58 enrolled. 35 intestinal ischemia, including 17 colonic.	Blood α-GST, Biotrin. Lactate.	On suspicion. Endoscopy, angiography or surgery. Four deaths excluded.	74% correctly classified. Combined-test Se 97–100%. Stand-alone Se/Sp NR.
S03. Block 2008 [26], Sweden	Prospective, *n* = 71. 10 infarctions of vascular/nonvascular origin.	Plasma D-dimer, α-GST, I-FABP and D-lactate. Immunoassay/enzymatic.	Within 24 h of pain. Mixed operative/imaging/clinical reference.	>0.3 mg/L, Se/Sp 100 (100–100)/44 (32–57)%. α-GST > 4 ng/mL, 20 (0–45)/87 (78–95)%. D-lactate >0.20 mmol/L, 90 (71–100)/23 (12–34)%.
S04. Polk 2008 [27], USA	Prospective surgical cohort, *n* = 26. 12 ischemia.	Serum IMA, cobalt-binding assay.	≤1 h before laparotomy. Operative classification.	≥0.35 absorbance units. Se/Sp 100/85.7%. AUC 0.946. Predictive-value labels conflict.
S05. Akyildiz 2009 [28], Turkey	Prospective selected cohort, *n* = 47. 28 AMI, 23 with necrosis.	Plasma D-dimer, MDA immunofiltration.	Before CTA. Operation or clinical follow-up.	>3.17 μg FEU/mL. Reported Se/Sp 94.7/78.6%. AUC 0.93 (0.81–0.98). Percentages conflict with denominators.
S06. Chiu 2009 [29], Taiwan	Prospective consecutive suspected AMI, *n* = 67. 23 AMI.	Plasma D-dimer, automated latex assay.	Before CT. CTA, surgery/pathology or angiography.	1.0 μg FEU/mL. Se/Sp 96/18%. AUC 0.64 (0.50–0.78). Existing hospital threshold.
S07. Thuijls 2011 [30], Netherlands	Prospective, *n* = 46. 22 intestinal ischemia. Ages 11–86. Urine *n* = 19.	Plasma/urine I-FABP, Hycult ELISA.	On suspicion. Histology or blinded clinical assessment.	Plasma 268 pg/mL, Se/Sp 68/71%, AUC 0.70 (0.53–0.86). Urine 551 pg/mL, 90/89%, AUC 0.93 (0.82–1.04).
S08. Kanda 2011 [31], Japan	Multicenter prospective, *n* = 361. 52 small-bowel ischemia, mostly strangulation. Ages 9–97.	Serum I-FABP, Osaka ELISA.	Within 24 h of diagnosis. Surgery or clinical recovery.	3.1 ng/mL. Se/Sp 78.8 (67.7–89.9)/73.8 (68.9–78.7)% in 361. AUC 0.792 in surgical subset *n* = 242.
S09. Matsumoto 2014 [32], Japan	Prospective CT-selected adults, *n* = 208. 24 vascular, 62 nonvascular ischemia.	Serum I-FABP, Osaka ELISA. D-dimer/lactate.	Initial clinical/CT assessment. Operation, pathology, autopsy or clinical course.	I-FABP 9.1 ng/mL, Se/Sp 83.3/89.1%, AUC 0.88 (0.79–0.96). Overlaps S22.
S10. Güzel 2014 [33], Turkey	Case–control, *n* = 77. 30 AMI, 27 other acute abdomen, 20 healthy.	Serum I-FABP, Hycult. Plasma D-dimer.	Presentation. AMI verified at surgery/pathology.	AMI versus healthy, I-FABP >90 pg/mL, Se/Sp 90/100%. D-dimer >130 μg/L, 93/100%.
S11. Gün 2014 [34], Turkey	Geriatric acute abdomen, *n* = 676. D-dimer tested in 230; 13 AMI.	Plasma D-dimer, Trinity latex assay.	Emergency evaluation. Selective CT/surgery.	>470 ng/mL. Se/Sp 84.6/47.9%. Selective testing/verification.
S12. van der Voort 2014 [35], Netherlands	Prospective ICU, *n* = 120. Definite diagnosis in 44, 23 ischemia.	Plasma I-FABP, Hycult. Enzymatic D-/L-lactate.	On suspicion. Mixed clinical/operative reference.	L-lactate 2.2 mmol/L in 44, Se/Sp 78/48%, AUC 0.65 (0.49–0.81). I-FABP/D-lactate nondiscriminatory.
S13. Shi 2015 [36], China	Prospective acute abdomen, *n* = 272. 39 mixed ischemia. Ages 8–87.	Serum I-FABP and D-lactate ELISA.	Before treatment. Surgery, imaging or clinical assessment.	I-FABP Se/Sp 76.2 (67.4–91.5)/74.8 (68.7–82.4)%, AUC 0.85. D-lactate 66.7 (52.8–84.2)/85.9 (77.8–92.6)%, AUC 0.69. Conflicting cutoffs.
S14. Klingele 2015 [37], Germany	Prospective elective cardiac surgery, *n* = 865. 78 angiographic NOMI.	Serum PCT, Elecsys BRAHMS.	Preoperative and next morning. Selective mesenteric angiography.	PCT 6.6 ng/mL, Se/Sp 71/94%, AUC 0.94 (0.92–0.96). Predictive postoperative target.
S15. Cossé 2015 [38], France	Retrospective operated ischemia, *n* = 128. 94 necrosis, 34 nonnecrotic injury.	Serum PCT, Kryptor TRACE.	Preoperative. Resection histology.	Necrosis ≥2.473 ng/mL, Se/Sp 94.6/68%, AUC 0.92 (0.86–0.98). No AMI present.
S16. Salim 2017 [39], Canada	Prospective operative/endoscopic cohort, *n* = 18. 13 with mixed ischemia.	Serum/urine I-FABP, R&D ELISA. Lactate.	≤1 h before intervention, 6/24 h after. Surgery/histology or endoscopy.	Urine 2.52 ng/mL, Se/Sp 92/80%, AUC 0.88. Serum AUC 0.66.
S17. Ludewig 2017 [40], Germany	Prospective ICU, *n* = 43. 21 ischemia. Urine *n* = 36.	Serum/urine I-FABP, Hycult ELISA.	On suspicion and serially. Operation/histology versus survival.	Serum 410.3 pg/mL, Se/Sp 33.3/95.5%, AUC 0.565. Urine 402.2 pg/mL, 81.3/70%, AUC 0.694.
S18. Kulu 2017 [41], Turkey	Prospective surgical comparison, *n* = 48. 23 AMI.	Plasma citrulline, chromatography. D-dimer/lactate.	Before operation. Operative reference.	Citrulline < 15.82 μmol/L, Se/Sp 39.13/100%, AUC 0.72 (0.57–0.84). Three-marker model AUC 0.90 (0.78–0.97), developed in the same sample.
S19. Nuzzo 2017 [42], France	Prospective confirmed AMI, *n* = 67. 23 irreversible transmural necrosis.	Routine blood L-lactate.	Clinical course to resection/follow-up. Histology, CT or recovery.	>2 mmol/L associated with necrosis. AUC 0.936 (0.866–0.997) belongs to a three-factor score.
S20. Zogheib 2018 [43], France	Retrospective matched cardiac surgery, *n* = 144. 48 intestinal ischemia.	Routine PCT/lactate, PALM score.	Postoperative serial testing. CT, endoscopy or surgery.	PCT > 4.7 μg/L, Se/Sp 85.42 (72.2–93.9)/84.37 (75.5–91.0)%. PALM ≥14, AUC 0.93 (0.91–0.95). Internal bootstrap only.
S21. Schellekens 2018 [44], Netherlands	Translational association. 22 ischemia, 40 acute-abdomen and 50 healthy controls.	Plasma SM22 and I-FABP, in-house ELISAs.	Clinical sampling. Operative/histological depth.	SM22 mean ± SEM 3.72 ± 0.91 ng/mL in 13 transmural cases versus 0.45 ± 0.08 in nine mucosal cases. No diagnostic threshold, Se/Sp or AUC.
S22. Matsumoto 2019 [23], Japan	Prospective CT-selected NOMI, *n* = 96. 25 NOMI.	Serum I-FABP, Osaka. D-dimer/lactate.	Initial clinical/CT assessment. Operation/autopsy or clinical course.	I-FABP 8.3 ng/mL, Se/Sp 76/80.3%, AUC 0.805 (0.696–0.914). Extension of S09, not independent.
S23. Mothes 2021 [45], Germany	Retrospective matched cardiac surgery, *n* = 432. 108 subsequent ischemia.	Routine blood L-lactate.	Preoperative, end of surgery, day-one mean. Ischemia within 14 days.	Day-one >3 mmol/L, Se/Sp 46.7/90.3%, AUC 0.68. OR 7.85 (4.62–13.3). Time-point *n* varies.
S24. Nuzzo 2021 [46], France	Prospective referral comparison, *n* = 129. 50 arterial/venous AMI, 79 symptomatic controls.	Plasma I-FABP ELISA, citrulline UPLC-MS, enzymatic D-lactate.	Admission. CTA/multidisciplinary diagnosis.	Citrulline < 16.6 μmol/L, Se/Sp 56/84%, AUC 0.679 (0.577–0.781). Source table: I-FABP AUC 0.401 (0.291–0.512), D-lactate 0.438 (0.328–0.547).
S25. Collange 2022 [47], France	Retrospective matched ICU, *n* = 274. 137 AMI.	Routine serum/blood L-lactate.	Highest in 0–24/24–48 h. CT, endoscopy or surgical reports.	Day-zero ≥ 2.0 mmol/L, Se/Sp 72 (64–80)/43 (34–51)%, AUC 0.57 (0.51–0.64). Day-one AUC 0.60 (0.53–0.67).
S26. Bourcier 2022 [48], France	Prospective DIAGOMI ICU, *n* = 61. Necrosis comparison, *n* = 40, 27 positive.	Plasma I-FABP, R&D. Chromatographic citrulline.	First confirmatory examination. CT/endoscopy/surgery, three probable cases.	Necrosis I-FABP 3114 pg/mL, Se/Sp 70/85%, AUC 0.83 (0.70–0.96). Citrulline not discriminative.
S27. Straarup 2024 [49], Denmark	Prospective surgical cohort, *n* = 1479 analyzed. 23 mixed target-positive, only six vascular necrosis.	Plasma D-lactate, Megazyme/Cobas.	Referral/suspicion. Operation or clinical assessment.	≥0.0925 mmol/L. Se/Sp 26.1/89.2%, AUC 0.588 (0.465–0.712). 1479 lipemic samples excluded.
S28. Acosta 2024 [50], International, 32 hospitals	AMESI prospective source cohort, *n* = 360. 73 MVT. D-dimer *n* = 57.	Plasma D-dimer, multiple local assays. Lactate.	Clinical admission/suspicion. CT or other clinical reference.	Above local normal range, Se/Sp 100 (85.2–100)/20.6 (8.7–37.9)%, AUC 0.63 (0.49–0.78). Different ROC-optimal cutoff: 5 mg/L.
S29. Taghiyev 2025 [51], Germany	Prospective cardiac-surgery registry, *n* = 500. Biomarker subset 48, six confirmed.	Serum I-FABP, Hycult. Routine lactate.	Five perioperative points to 36 h. Operation/histology or treated NOMI.	At 36 h ≥ 1421 pg/mL, reported Se/Sp 100/91.9%. Source AUCs 0.973 (0.764–0.997) and 0.779 (0.295–0.945), contrast unclear.

Se/Sp, sensitivity/specificity in percent. AUC, area under the receiver operating characteristic curve. PCT, procalcitonin. MVT, mesenteric venous thrombosis. NR, not reported in the source accessed. S01–S29 identify publications, not independent cohorts. Values are reported at their own thresholds, targets and tested denominators. S21 is an association report. S09 and S22 overlap. CI, confidence interval. Parentheses after diagnostic estimates denote published 95% CIs where available. Published bounds are reproduced without recalculation or truncation. Where no interval is shown, source precision could not be established for that displayed estimate. OR, odds ratio. SEM, standard error of the mean.

**Table 3:** QUADAS-2 judgments for the primary-report inventory.

Report	Patient Selection	Index Test	Reference	Flow/Timing	Applicability Patients	Applicability Index	Applicability Target
Delaney 1999 [24]	U	H	U	H	H	L	H
Gearhart 2003 [25]	U	U	U	H	H	U	H
Block 2008 [26]	L	H	U	H	H	L	H
Polk 2008 [27]	H	H	U	L	H	L	H
Akyildiz 2009 [28]	H	H	U	H	H	L	L
Chiu 2009 [29]	L	U	U	H	L	L	L
Thuijls 2011 [30]	L	H	L	H	H	L	H
Kanda 2011 [31]	U	H	H	H	H	L	H
Matsumoto 2014 [32]	H	H	U	H	H	L	L
Güzel 2014 [33]	H	H	U	H	H	L	L
Gün 2014 [34]	H	U	U	H	H	L	L
van der Voort 2014 [35]	L	H	H	H	H	L	H
Shi 2015 [36]	U	H	U	H	H	U	H
Klingele 2015 [37]	L	H	L	H	H	L	H
Cossé 2015 [38]	H	H	U	L	H	L	H
Salim 2017 [39]	H	H	U	H	H	L	H
Ludewig 2017 [40]	H	H	H	H	H	L	H
Kulu 2017 [41]	H	H	U	L	H	U	L
Nuzzo 2017 [42]	L	U	U	H	H	H	H
Zogheib 2018 [43]	H	H	U	H	H	H	H
Schellekens 2018 [44]	NA	NA	NA	NA	NA	NA	NA
Matsumoto 2019 [23]	H	H	U	H	H	L	L
Mothes 2021 [45]	H	U	U	H	H	H	H
Nuzzo 2021 [46]	H	H	L	H	U	L	L
Collange 2022 [47]	H	H	U	H	H	H	U
Bourcier 2022 [48]	U	H	H	H	H	L	H
Straarup 2024 [49]	U	H	U	H	H	L	H
Acosta 2024 [50]	U	L	U	H	L	L	L
Taghiyev 2025 [51]	H	H	U	H	H	H	H

H, high risk/concern. L, low risk/concern. U, unclear. NA, not assigned for S21, which did not report diagnostic performance. *N* = 28 for diagnostic domain summaries. Multi-test judgments refer to the displayed diagnostic contrast. The S03 index-test rating concerns exploratory D-dimer thresholds, while α-GST and D-lactate had prespecified cutoffs. For Acosta 2024 (S28), the displayed rating concerns existing assay normal limits [50]. The separate ROC-derived 5 mg/L cutoff has a high index-test risk. No numerical overall score was calculated.

**Table 4:** Context from published diagnostic-performance syntheses.

Marker or Assay	Sensitivity, % (95% CI)	Specificity, % (95% CI)	Source and Interpretation
I-FABP, Uden assay	79.0 (66.5–88.5)	91.3 (87.0–94.6)	Treskes et al. [11]. Assay-specific pooled result.
I-FABP, Osaka assay	75.0 (67.9–81.2)	79.2 (76.2–82.0)	Treskes et al. [11]. Separate analytical platform.
α-GST	67.8 (54.2–79.5)	84.2 (75.3–90.9)	Treskes et al. [11]. Limited clinical evidence.
IMA	94.7 (74.0–99.9)	86.4 (65.1–97.1)	Treskes et al. [11]. Wide intervals from a small evidence base.
D-lactate	71.7 (58.6–82.5)	74.2 (69.0–79.0)	Treskes et al. [11]. Heterogeneous intestinal-ischemia populations.
Serum I-FABP, overall	73.9	90.5	Reintam Blaser et al. [12]. Published pool of 16 studies across types and stages. Not a stage-specific estimate.
D-dimer, 2013 synthesis	96 (89–99)	40 (33–47)	Cudnik et al. [52]. High sensitivity with many false positives.
L-lactate, 2013 synthesis	86 (73–94)	44 (32–55)	Cudnik et al. [52]. Does not establish reliable exclusion of early AMI.
D-dimer, 2025 synthesis	85 (80–89)	50 (46–54)	Alenazi Abdulmajeed Farhan and Alanazi Saleh Farhan [53]. Not an independent validation of the older pool.
Lactate, 2025 synthesis	67 (57–76)	67 (58–75)	Same synthesis [53]. Different evidence set and study methods.

CI, confidence interval, shown where reproduced. Values are published secondary estimates. Underlying studies and cohorts may overlap. The IMA pool includes clinical evidence outside the 29-report set. No row defines a validated AMI threshold.

**Table 5:** Biological properties and candidate injury signals.

Marker	Molecular Property	Biological Source and Candidate Signal	Specimen	Typical Analytical Method
I-FABP	Small cytosolic fatty acid-binding protein	Small-intestinal enterocytes. Mucosal injury signal.	Blood and urine	Research immunoassay
α-GST	Cytosolic detoxification enzyme	Enterocytes and other tissues, including liver. Cellular injury signal.	Blood	Isoenzyme-specific immunoassay
Citrulline	Non-proteinogenic amino acid	Intestinal synthesis and renal metabolism. Enterocyte function.	Blood	Amino-acid chromatography or mass spectrometry
SM22	Smooth-muscle-associated protein	Intestinal and other smooth muscle. Candidate deeper mural injury signal.	Blood	Research immunoassay
D-dimer	Cross-linked fibrin degradation product	Fibrin turnover. Coagulation context.	Blood	Automated immunoassay
Procalcitonin	Peptide precursor of calcitonin	Systemic inflammation. Candidate inflammatory response signal.	Blood	Automated immunoassay
IMA	Albumin with altered metal-binding properties	Altered albumin metal binding. General ischemic stress.	Blood	Albumin cobalt-binding assay or related methods
L-lactate	Predominant physiological lactate stereoisomer	Production and clearance during metabolic stress. Metabolic severity.	Blood	Enzymatic analysis or blood-gas analyzer
D-lactate	Stereoisomer linked to microbial metabolism	Microbial metabolism and host clearance. Candidate barrier-associated signal.	Blood	Stereospecific enzymatic or specialist assay

Characteristics are summarized from the clinical and mechanistic literature cited in Section 3.3, Section 3.4 and Section 3.5. Candidate injury signals are hypothesis-generating and unvalidated. Assays and local availability vary.

**Table 6:** GRADE summary for defined diagnostic questions.

Marker/Report Count	Risk of Bias	Inconsistency	Indirectness	Imprecision	Publication Bias	Diagnostic Certainty
Blood I-FABP 13 reports	Very serious (−2)	Serious (−1)	Serious (−1)	Serious (−1)	Unclear, 0	Very low
Urine I-FABP 3 reports	Very serious (−2)	Serious (−1)	Serious (−1)	Very serious (−2)	Unclear, 0	Very low
α-GST 3 reports	Very serious (−2)	Serious (−1)	Serious (−1)	Serious (−1)	Unclear, 0	Very low
Citrulline 3 reports	Very serious (−2)	Serious (−1)	Serious (−1)	Serious (−1)	Unclear, 0	Very low
D-dimer 9 reports	Very serious (−2)	Serious (−1)	Serious (−1)	Serious (−1)	Unclear, 0	Very low
L-lactate 12 reports	Very serious (−2)	Serious (−1)	Very serious (−2)	Serious (−1)	Unclear, 0	Very low
D-lactate 5 reports	Very serious (−2)	Serious (−1)	Serious (−1)	Serious (−1)	Unclear, 0	Very low
Procalcitonin 3 reports	Very serious (−2)	Serious (−1)	Very serious (−2)	Serious (−1)	Unclear, 0	Very low
IMA 1 report	Very serious (−2)	Not assessable	Serious (−1)	Very serious (−2)	Unclear, 0	Very low
SM22 1 report	Not assessed	Not assessed	Not assessed	Not assessed	Not assessed	Not estimable

Ratings start from a potentially high-certainty diagnostic evidence body. Downgrades are shown explicitly and certainty is bounded at very low. Inconsistency cannot be assessed for a single IMA report. SM22 has no diagnostic estimate to grade. Publication bias is undetermined and receives no automatic downgrade. Counts are reports addressing the marker, not independent cohorts or a summed patient population. Blood/urine I-FABP reports overlap.

**Table 7:** Clinical interpretation of biomarker results.

Marker	Potential Information	Current Limitation
I-FABP	Enterocyte injury	Cannot establish vascular etiology, exclude AMI, or determine transmurality.
α-GST	Cellular injury	Incomplete tissue specificity and insufficient sensitivity for exclusion.
Citrulline	Functional enterocyte mass	No validated acute rule-out threshold.
SM22	Possible deeper mural injury	Cannot determine resection extent or replace assessment of viability.
D-dimer	Fibrin turnover	An elevated result is nonspecific. A negative result cannot replace an indicated CTA.
L-lactate	Metabolic disturbance and response to resuscitation	May be normal early and cannot localize the cause.
D-lactate	Microbial metabolic signal	Limited validation and specialist analysis.
Procalcitonin	Inflammatory or infectious context	Does not localize injury to the intestine.
IMA	General ischemic or oxidative stress	No established intestinal specificity.

Interpretation is based on the evidence discussed above. No biomarker result should delay CTA when AMI is suspected [2].

**Table 8:** Practical availability and readiness for AMI assessment.

Test Group	Access in Practice	Requirements for an AMI Application
L-lactate	Common emergency and blood-gas platforms	Interpret with perfusion, clearance, treatment, and timing.
D-dimer	Routine coagulation laboratories	A defined AMI population, assay-specific units, threshold validation, and acceptable false-negative risk.
Procalcitonin	Automated platforms in many hospitals	Separate infection and systemic inflammation from intestinal injury.
Citrulline	Specialist amino-acid analysis	Emergency turnaround, renal-function adjustment, and prospective validation.
D-lactate	Specialist testing with variable access	Stereospecific analysis and a clinically relevant turnaround. Routine lactate assays cannot be assumed to measure it.
I-FABP, α-GST, and SM22	Predominantly research assays in AMI studies	Analytical harmonization, appropriate reference materials, validated thresholds, and rapid testing.
IMA	Assay access is laboratory-dependent	Reproducible methods and demonstration of added clinical value. No established routine AMI role.
Urinary I-FABP and PGE-MUM	Research use for AMI	Control of dilution and renal effects. Human diagnostic validation is especially lacking for PGE-MUM.

Availability varies by institution and jurisdiction. This is a practical classification, not a statement of regulatory approval. Clinical validation and access issues are described in the BIPAMI and TACTIC protocols [59,60].

**Table 9:** Implementation barriers and corresponding validation tasks.

Barrier	Consequence	Required Work
Undefined intended use	The same assay is asked to detect AMI, determine viability, and predict outcome.	Specify one clinical decision, target population, and acceptable error rate.
Assay variation	A threshold from one platform may not transfer to another.	Document calibration, precision, matrix effects, sample stability, and interlaboratory agreement.
Slow or restricted access	Results arrive after imaging or treatment.	Measure real collection-to-result time and assess a rapid platform.
Unproven incremental value	Statistical association does not show a change in care.	Compare a clinical model with and without the marker at the intended decision point.
Limited clinical validation	False reassurance or unnecessary escalation can follow.	Use independent cohorts with prespecified thresholds and adjudicated outcomes.
Regulatory and economic requirements	A research assay may be unsuitable for routine service.	Verify the intended clinical indication, local regulatory requirements, workload, and cost.

These are proposed implementation tasks. The protocols cited above illustrate the need for coordinated sampling, centralized analysis, and clinical adjudication [59,60].

**Table 10:** Sources of heterogeneity and their effects on comparisons.

Dimension	Typical Difference	Effect on Interpretation
Patient selection	Consecutive suspected cases versus confirmed disease and healthy controls	Different clinical spectra alter discrimination, predictive values, and applicability.
AMI subtype and severity	Arterial, venous, and non-occlusive disease. Mucosal injury versus necrosis.	A marker may reflect one mechanism or injury burden. A pooled value may hide this dependence.
Reference standard	CTA, surgery, histopathology, or a composite assessment	These measure vascular abnormalities, operative viability, or tissue injury at different times. Verification may depend on disease severity.
Assay and specimen	Different antibodies, calibrators, matrices, units, and storage conditions	Numerically similar cutoffs may not represent equivalent concentrations or analytical behavior.
Sampling time	Before or after resuscitation, reperfusion, operation, or symptom progression	Release and clearance change over time. A later sample may appear more discriminating because disease has advanced.
Diagnostic threshold	Prespecified cutoff versus one optimized in the same cohort	Post hoc optimization can overestimate performance and impair external transfer.

The clinical implications are informed by published systematic assessments and assay-specific findings [11,12,17]. These differences support stratified interpretation rather than direct ranking of pooled estimates.

**Table 11:** Challenges in developing evidence for new biomarkers.

Challenge	Risk to the Evidence	Design Response
Relatively few eligible cases at each center	Small samples and unstable thresholds	Multicenter enrollment with a screening log and realistic recruitment targets.
Urgent care and uncertain onset	Missing early samples and inaccurate timing	Embed sampling in routine care and record actual intervals without delaying treatment.
Unequal verification	Surgical cases receive histology while noncases have imaging or follow-up	Prespecify adjudication and follow-up. Report the reference standard used for each group.
Mixed mechanisms and injury depth	An overall result masks clinically different subgroups	Record subtype, extent, and depth with prespecified subgroup questions.
Many candidate markers and few outcomes	Overfitting and selective reporting	Limit predictors, justify sample size, prespecify analyses, and report all tested markers.
No independent validation	Optimistic local performance	Separate model development from external validation in time or location.

These design proposals reflect the clinical problem and the practical considerations described in the BIPAMI and TACTIC protocols [59,60].

## Data Availability

The data supporting this review are included in the article. Source publications are identified in the reference list.

## Supplementary Materials

The following supporting information can be downloaded at: https://www.mdpi.com/article/10.3390/diagnostics16203360/s1.
