# Supplementary material for: Biomarkers in Acute Mesenteric Ischemia: A Systematic Review of Diagnostic Performance and Injury-Related Signals
Source: Diagnostics (Basel). 2026 Oct 15;16(20):3360. doi: 10.3390/diagnostics16203360 (PMC13703922; doi:10.3390/diagnostics16203360)
Supplement: Supplementary file 1 [file diagnostics-16-03360-s001.zip › diagnostics-4546804-supplementary.pdf]

PRISMA 2020 Checklist

Manuscript diagnostics-4546804

Section and Topic	Item #	Checklist item	Location where item is reported
**TITLE**			
Title	1	Identify the report as a systematic review.	Title on the first page.
**ABSTRACT**			
Abstract	2	See the PRISMA 2020 for Abstracts checklist.	Abstract. See the completed abstract checklist below.
**INTRODUCTION**			
Rationale	3	Describe the rationale for the review in the context of existing knowledge.	Section 1, paragraphs on clinical need, existing reviews and biomarker limitations.
Objectives	4	Provide an explicit statement of the objective(s) or question(s) the review addresses.	Section 1, final paragraph.
**METHODS**			
Eligibility criteria	5	Specify the inclusion and exclusion criteria for the review and how studies were grouped for the syntheses.	Sections 2.3 and 2.7.
Information sources	6	Specify all databases, registers, websites, organisations, reference lists and other sources searched or consulted to identify studies. Specify the date when each source was last searched or consulted.	Section 2.2. Searches were conducted on 10 October 2026. Citation searches used the 29 included reports and two reviews, Treskes et al. [11] and Reintam Blaser et al. [12].
Search strategy	7	Present the full search strategies for all databases, registers and websites, including any filters and limits used.	Section 2.2. Full PubMed query, Cochrane Search Manager lines and citation-search approach.
Selection process	8	Specify the methods used to decide whether a study met the inclusion criteria of the review, including how many reviewers screened each record and each report retrieved, whether they worked independently, and if applicable, details of automation tools used in the process.	Section 2.4. Two independent screeners and agreed eligibility decisions.
Data collection process	9	Specify the methods used to collect data from reports, including how many reviewers collected data from each report, whether they worked independently, any processes for obtaining or confirming data from study investigators, and if applicable, details of automation tools used in the process.	Section 2.5. Two researchers (S.N. and M.I.) independently extracted data from each included report.
Data items	10a	List and define all outcomes for which data were sought. Specify whether all results that were compatible with each outcome domain in each study were sought (e.g. for all measures, time points, analyses), and if not, the methods used to decide which results to collect.	Section 2.5. Clinical targets, reported measures, time points and selection of principal comparisons.
	10b	List and define all other variables for which data were sought (e.g. participant and intervention characteristics, funding sources). Describe any assumptions made about any missing or unclear information.	Section 2.5. Study and test variables, missing information, conflicts and cohort overlap.
Study risk of bias assessment	11	Specify the methods used to assess risk of bias in the included studies, including details of the tool(s) used, how many reviewers assessed each study and whether they worked independently, and if applicable, details of automation tools used in the process.	Section 2.6. Two independent QUADAS-2 assessors, consensus and domain-based judgments.
Effect measures	12	Specify for each outcome the effect measure(s) (e.g. risk ratio, mean difference) used in the synthesis or presentation of results.	Section 2.5. Sensitivity, specificity, AUC, reported association estimates and published precision.

PRISMA

PRISMA 2020 Checklist

Section and Topic	Item #	Checklist item	Location where item is reported
Synthesis methods	13a	Describe the processes used to decide which studies were eligible for each synthesis (e.g. tabulating the study intervention characteristics and comparing against the planned groups for each synthesis (item #5)).	Sections 2.3 and 2.7. Grouping by marker, specimen and clinical target.
	13b	Describe any methods required to prepare the data for presentation or synthesis, such as handling of missing summary statistics, or data conversions.	Section 2.5. Missing values, reconstructable data, separate thresholds and overlapping reports.
	13c	Describe any methods used to tabulate or visually display results of individual studies and syntheses.	Section 2.7. Chronological tables, individual and aggregate domain displays, GRADE summary.
	13d	Describe any methods used to synthesize results and provide a rationale for the choice(s). If meta-analysis was performed, describe the model(s), method(s) to identify the presence and extent of statistical heterogeneity, and software package(s) used.	Section 2.7. Narrative synthesis and reasons for not pooling.
	13e	Describe any methods used to explore possible causes of heterogeneity among study results (e.g. subgroup analysis, meta-regression).	Section 2.7. Descriptive comparison across six sources of heterogeneity.
	13f	Describe any sensitivity analyses conducted to assess robustness of the synthesized results.	Section 2.7. No formal sensitivity analysis was conducted.
Reporting bias assessment	14	Describe any methods used to assess risk of bias due to missing results in a synthesis (arising from reporting biases).	Section 2.7. Formal reporting-bias assessment and small-study-effect tests were not performed.
Certainty assessment	15	Describe any methods used to assess certainty (or confidence) in the body of evidence for an outcome.	Section 2.7. Diagnostic GRADE domains, starting certainty and downgrade rules.
**RESULTS**			
Study selection	16a	Describe the results of the search and selection process, from the number of records identified in the search to the number of studies included in the review, ideally using a flow diagram.	Section 3.1 and Figure 1. Selection counts and exclusion criteria are reported. Counts for individual exclusion reasons are not reported. Cohort overlap is described in Section 3.1.
	16b	Cite studies that might appear to meet the inclusion criteria, but which were excluded, and explain why they were excluded.	Section 3.1. Cited contextual reports excluded from primary diagnostic evidence, with reasons.
Study characteristics	17	Cite each included study and present its characteristics.	Section 3.1 and Table 2. All 29 reports are cited and characterized.
Risk of bias in studies	18	Present assessments of risk of bias for each included study.	Section 3.2, Table 3 and Figures 2–3. Report-level QUADAS-2 judgments and domain summaries.
Results of individual studies	19	For all outcomes, present, for each study: (a) summary statistics for each group (where appropriate) and (b) an effect estimate and its precision (e.g. confidence/credible interval), ideally using structured tables or plots.	Table 2 and Sections 3.3–3.5. Study-level estimates and published CIs or dispersion where available.
Results of syntheses	20a	For each synthesis, briefly summarise the characteristics and risk of bias among contributing studies.	Sections 3.2–3.7 and Tables 2, 3 and 6.

PRISMA

PRISMA 2020 Checklist

Section and Topic	Item #	Checklist item	Location where item is reported
	20b	Present results of all statistical syntheses conducted. If meta-analysis was done, present for each the summary estimate and its precision (e.g. confidence/credible interval) and measures of statistical heterogeneity. If comparing groups, describe the direction of the effect.	Section 2.7. No new statistical synthesis. Published pools: Section 3.6 and Table 4.
	20c	Present results of all investigations of possible causes of heterogeneity among study results.	Sections 3.3–3.7 and 4.6, with Table 10. Descriptive heterogeneity findings.
	20d	Present results of all sensitivity analyses conducted to assess the robustness of the synthesized results.	No formal sensitivity analysis. See Section 2.7.
Reporting biases	21	Present assessments of risk of bias due to missing results (arising from reporting biases) for each synthesis assessed.	Section 3.7 and Table 6. Reporting bias remains undetermined.
Certainty of evidence	22	Present assessments of certainty (or confidence) in the body of evidence for each outcome assessed.	Section 3.7 and Table 6, with domain-specific reasons for downgrading.
**DISCUSSION**			
Discussion	23a	Provide a general interpretation of the results in the context of other evidence.	Sections 4.1–4.5 and 5.
	23b	Discuss any limitations of the evidence included in the review.	Section 4.6, supported by Sections 3.2–3.7.
	23c	Discuss any limitations of the review processes used.	Section 4.6. Protocol, search coverage, source reporting, overlap and language restrictions.
	23d	Discuss implications of the results for practice, policy, and future research.	Sections 4.4–4.7 and 5, with Tables 7–11 and Figure 6.
**OTHER INFORMATION**			
Registration and protocol	24a	Provide registration information for the review, including register name and registration number, or state that the review was not registered.	Section 2.1 and Abstract. The review was not registered in PROSPERO.
	24b	Indicate where the review protocol can be accessed, or state that a protocol was not prepared.	Section 2.1. No review protocol was prepared.
	24c	Describe and explain any amendments to information provided at registration or in the protocol.	Not applicable. No review protocol was prepared and the review was not registered (Section 2.1).
Support	25	Describe sources of financial or non-financial support for the review, and the role of the funders or sponsors in the review.	Funding and Author Contributions. Support from coauthors E. Klimova and D. Pogosyan and their roles.
Competing interests	26	Declare any competing interests of review authors.	Conflicts of Interest statement.
Availability of data, code and other materials	27	Report which of the following are publicly available and where they can be found: template data collection forms; data extracted from included studies; data used for all analyses; analytic code; any other materials used in the review.	Data Availability; Tables 2, 3 and 6; Section 2.2. No pooling code was used.

From: Page MJ, McKenzie JE, Bossuyt PM, Boutron I, Hoffmann TC, Mulrow CD, et al. The PRISMA 2020 statement: an updated guideline for reporting systematic reviews. BMJ 2021;372:n71. doi: 10.1136/bmj.n71. This work is licensed under CC BY 4.0. To view a copy of this license, visit https://creativecommons.org/licenses/by/4.0/

Locations refer to section and table numbers in the accompanying manuscript. Unreported procedural details are identified explicitly.

PRISMA 2020 Checklist

PRISMA 2020 for Abstracts

Item	Reporting requirement	Location and coverage
1	Identify a systematic review	Title on the first page.
2	State the main question	Abstract, Background/Objectives.
3	State eligibility criteria	Abstract, Methods. Clinical reports, languages and minimum sample size.
4	Name sources and search dates	Abstract, Methods. PubMed/MEDLINE, Cochrane Library and citation links, 10 October 2026. The PubMed and citation-search publication limit was October 2026.
5	Describe risk-of-bias appraisal	Abstract, Methods. QUADAS-2.
6	Describe the synthesis	Abstract, Methods. Narrative synthesis with diagnostic GRADE.
7	Report included evidence and sample size	Abstract, Results. 29 reports and cohort range 18–1479. A unique participant total cannot be established because cohorts overlap and tested denominators vary.
8	Report principal findings	Abstract, Results. Domain-risk counts, very low certainty and marker-specific findings. No new meta-analysis.
9	State the main evidence limitations	Abstract, Results. High risk of bias, imprecision and dependence on population, assay and timing.
10	Give the interpretation and implications	Abstract, Conclusions. No stand-alone rule-out test, panel validation and unvalidated models.
11	Name the principal financial support	Abstract, Funding. Ekaterina Klimova and David Pogosyan.
12	State registration status	Abstract, Registration. Review not registered.

Based on the PRISMA 2020 for Abstracts checklist accompanying Page et al., BMJ 2021;372:n71, doi:10.1136/bmj.n71. Reporting requirements above are abbreviated.
